# The Women of FOCIS: Promoting Equality and Inclusiveness in a Professional Federation of Clinical Immunology Societies

**DOI:** 10.3389/fimmu.2022.816535

**Published:** 2022-04-04

**Authors:** Elaine F. Reed, Anita S. Chong, Megan K. Levings, Caley Mutrie, Terri M. Laufer, Maria Grazia Roncarolo, Megan Sykes

**Affiliations:** ^1^ UCLA Immunogenetics Center, Department of Pathology and Laboratory Medicine, University of California, Los Angeles, Los Angeles, CA, United States; ^2^ Section of Transplantation, Department of Surgery, University of Chicago, Chicago, IL, United States; ^3^ Department of Surgery, University of British Columbia, Vancouver, BC, Canada; ^4^ Federation of Clinical Immunology Societies, Menomonee Falls, WI, United States; ^5^ Division of Rheumatology, Department of Medicine, Perelman School of Medicine, University of Pennsylvania, Philadelphia, PA, United States; ^6^ Division of Hematology, Oncology, Stem Cell Transplantation, and Regenerative Medicine, Department of Pediatrics, Stanford University School of Medicine, Stanford, CA, NY, United States; ^7^ Columbia Center for Translational Immunology, Department of Medicine, Columbia University, New York, NY, United States

**Keywords:** gender, equality, professional society, career, leadership

## Abstract

The authors of this article, all women who have been deeply committed to the Federation of Clinical Immunology Societies (FOCIS), performed a retrospective analysis of gender equality practices of FOCIS to identify areas for improvement and make recommendations accordingly. Gender data were obtained and analyzed for the period from January 2010 to July 2021. Outcome measures included numbers of men and women across the following categories: membership enrollment, meeting and course faculty and attendees, committee and leadership composition. FOCIS’ past and present leaders, steering committee members, FCE directors, individual members, as well as education, annual meeting scientific program and FCE committee members and management staff of FOCIS were surveyed by email questionnaire for feedback on FOCIS policies and practice with respect to gender equality and inclusion. Although women represent 50% of the membership, they have been underrepresented in all leadership, educational, and committee roles within the FOCIS organization. Surveying FOCIS leadership and membership revealed a growing recognition of disparities in female leadership across all FOCIS missions, leading to significant improvement in multiple areas since 2016. We highlight these changes and propose a number of recommendations that can be used by FOCIS to improve gender equality.

## Introduction

Professional societies provide an important role in the advancement of science and member careers by promoting research, education, professional development and networking opportunities. To meet these objectives, societies provide a platform that enables members to communicate research findings, network with colleagues, and foster collaborations with academic partners, regulatory and funding agencies, biotech and pharma.

The mission of the federation of clinical immunology societies (FOCIS) is to improve human health through immunology by fostering interdisciplinary approaches to both understand and treat immune-based diseases. Through FOCIS, researchers and clinicians share knowledge across traditional disease borders, and identify commonalities between treatments and therapies that are life-changing for those impacted by immune-mediated diseases. Initially established as a cross-disciplinary meeting, FOCIS held its first Annual Meeting in 2001. FOCIS was incorporated as a 501(c)(3) organization in 2003. Today, FOCIS has 60 Member Societies (both in the United States and internationally), representing about 65,000 clinicians and scientists and 1,191 individual members worldwide. FOCIS continues to hold an annual meeting for scientific exchange, education, networking and building collaborations between researchers in academia and industry. FOCIS also sponsors educational courses held throughout the world.

The FOCIS governance structure consists of a board of directors and a steering committee comprised of 10 permanent and 10 rotating member societies. As is apparent from the data discussed below, as FOCIS expanded the number of member societies and created an individual membership category, there was a significant underrepresentation of women appointed to leadership positions in its first two decades. A leadership dominated by men did not represent its constituents, as women represented roughly 50% of membership and annual meeting attendance.

In association with growing recognition of the under-representation of women in FOCIS leadership, in 2016 the nominating committee nominated, and the steering committee elected, the first female president of FOCIS. Dr. Maria Grazia Roncarolo, the inaugural female president, assessed the gender composition of each FOCIS committee and proposed more women as board members and committee chairs and worked with the program committee to invite more female speakers to present at annual meetings. In a further development, in 2021, under the leadership of Drs. Megan Sykes and Mark Anderson, FOCIS established the Women in Clinical Immunology Committee (WICI) to foster understanding and awareness of gender issues, promote career development of women in immunology, and advance understanding of the role of sex and gender in immunological disease. Women in Clinical Immunology (WICI) has a mandate to support career development for women in immunology, to advocate for women’s participation and leadership at all levels in FOCIS and in the greater immunology community, and to promote the science of sex and gender in immunology.

As part of this process to improve gender equality, we have reviewed available data on the composition of the FOCIS board of directors, steering committee, membership, education, annual meeting scientific program and FOCIS centers of excellence (FCE) committees during an approximately 10-year period from January 2010 to July 2021. We also surveyed past presidents, directors, steering committee members, FCE directors, management staff and membership, education, annual scientific program and FCE committee members on their impression of gender equality in FOCIS over time. Here we review the composition of FOCIS, its leadership, committees and programs during the past decade in order to assess gender equality, identify areas for improvement and discuss actions to be taken.

## Methods

### Data Collection

Available data were analyzed for the period from January 2010 to July 2021. Data on gender was sourced from the FOCIS membership database and leadership and committee rosters. Meeting and event registrants, meeting and course speakers, chairs and faculty, and individual members and non-members self-report whether they identify as male, female or decline to report, when registering for meetings and related activities and applying for individual membership. Each response is stored in the FOCIS membership database. In some instances, FOCIS staff enters this information into the database on an individual’s behalf. Outcome measures included numbers of men and women across the following categories: membership enrollment, meeting and course faculty and attendance, committee and leadership composition and steering committee composition. This report is limited to analysis of males and females who reported their gender. Unreported/missing data was tabulated and summarized in **Supplement Table 1**. The data is available upon request by contacting Caley Mutrie (email address: cmutrie@focisnet.org).

### Surveys

FOCIS’s past and present leadership, steering committee members, senior and junior members, and management staff of FOCIS were surveyed by questionnaire for feedback on FOCIS policies and practice with respect to gender equality and inclusion. Anonymous surveys were distributed by email to 131 potential respondents. Participants included members of the board of directors, past presidents, steering committee members, FCE directors, individual members, education committee members, annual meeting scientific program and FCE committee members, and management staff. A 12-question survey was distributed (**Supplement Table 2**). The surveys included six closed-ended questions regarding impressions of FOCIS’s performance in gender equality during the period from the year of establishment to the present (2001-2021). Survey participants were asked to score performance of FOCIS on four dichotomous (yes/no) questions and three, five-point scale questions (two questions stating extremely important/important/somewhat important/slightly important/not at all important and one question stating extremely successful/very successful/somewhat successful/slightly successful/not at all successful). Five additional close-ended questions asked participants to specify gender, age, length of engagement with FOCIS, professional role and roles currently or previously held in FOCIS. Surveys also included one open-ended question for respondents to provide additional comments on gender equality in FOCIS and in immunology. FOCIS’s ten past and current presidents and president-elect were separately interviewed by email. The email questionnaire included two open-ended questions regarding their awareness of gender disparity in FOCIS prior to 2015 in the FOCIS leadership and education activities, and the events that eventually led to more inclusive representation after 2015. Permission to include quoted comments and opinions of the past and current presidents and president-elect was obtained.

### Statistical Analysis

To determine yearly changes in female participation in leadership and committee activities, data were analyzed as a function of gender (annual percentages of female respondents) regressed against time using a simple linear trend model. (Higher order temporal relationships, visually apparent in a few plots, were not analyzed due to the small number of respondents in the corresponding categories.) The results are displayed as pyramid plots.

## Results

### Women Comprise Half of the FOCIS Membership

**Figure 1:**
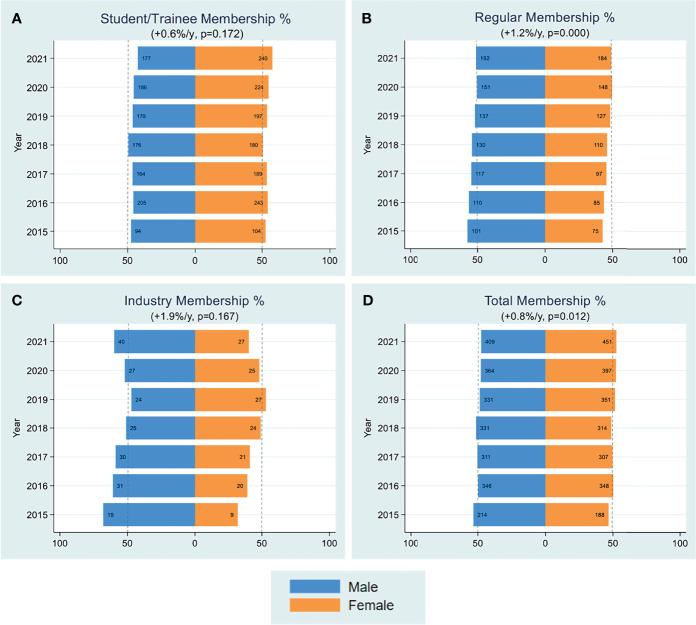
Membership. Yearly female percentages of student/trainee membership **(A)**, regular membership **(B)**, industry membership **(C)** and all categories combined **(D)** are shown over the period 2015-2021. Percentages were based on actual numbers subjects stating their gender. Average changes per year and p-values (shown in parentheses) were estimated by the slope coefficient from a simple linear regression model. The dotted line represents 50% as a point of reference for parity.

FOCIS established individual membership in 2015 that includes clinicians, researchers, industry experts, trainees and graduate and medical students. Currently, there are just under 1,200 FOCIS members. Member benefits include connecting to the global community of translational immunology leaders at the FOCIS annual meeting, a searchable membership directory, discounted educational offerings, eligibility for travel awards, free job postings on the career center, and free annual meeting abstract submission. Notably, women continue to represent greater than 50% of the membership of FOCIS (**Figure 1**). Total female membership and student and trainee membership has remained stable with an average 0.8% and 0.6% per year increase since 2015, respectively. Incremental increases in membership have occurred for women in the regular membership (+1.2%/year) and industry membership (+1.9%/year) categories.

### Disproportion of Women Planning, Chairing, and Speaking at FOCIS Annual Meetings and Educational Workshops

**Figure 2:**
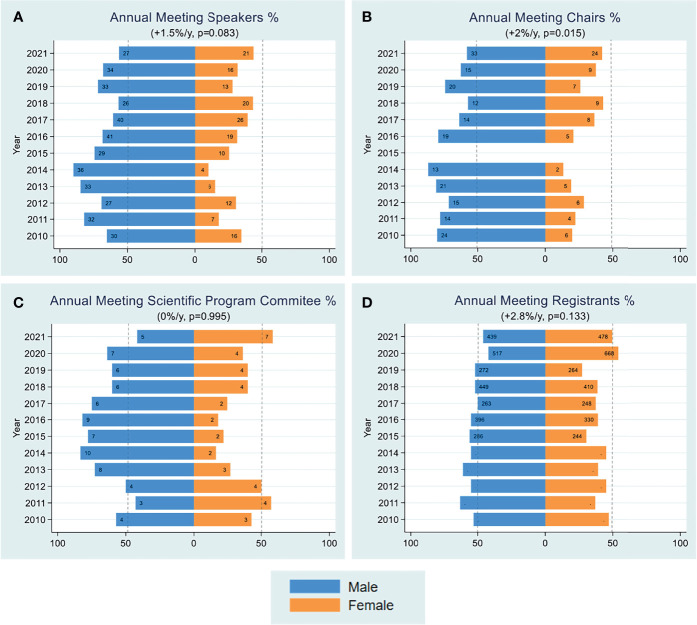
Annual Meeting. Yearly female percentages of annual meeting speakers **(A)**, session chairs **(B)**, scientific program committee **(C)**, and registrants **(D)** are shown over the period 2010-2021. Percentages were based on actual numbers subjects stating their gender. Average changes per year and p-values (shown in parentheses) were estimated by the slope coefficient from a simple linear regression model. Gender data was not collected for annual meeting chairs in 2015. The dotted line represents 50% as a point of reference for parity.

Female registrants for FOCIS annual meetings over time are shown in **Figure 2**. Average changes in female meeting registrants increased from 40% in 2010 to 50% in 2021 at a rate of 1% per year. In sharp contrast, women were severely underrepresented as scientific program planners, session chairs and invited speakers. From 2010-2015 women represented less than 25% of invited speakers. This disparity improved somewhat over time, with an average 1.5% per year increase in female speakers by 2021, but nonetheless, men still represented 60% of the invited speakers at the annual FOCIS meeting. The most significant increase was in the percentage (2%/year) of women chairing sessions at the FOCIS annual meeting, yet they still only represented 20-40% of session chairs in a given year.

The percentage of women serving on the annual meeting scientific program committee was only about 20% during the period from 2013-2017, increased to 36-40% in the next three years and the 2021 program committee includes 58% women (**Figure 2**). Given that the program committee chooses the plenary and thematic sessions and speakers, it was vital to appoint women to the program committee to achieve gender parity, dissolve stereotypes, provide role models and improve the meeting quality and attendance; the increasing trend of women representation in this committee is encouraging.

**Figure 3:**
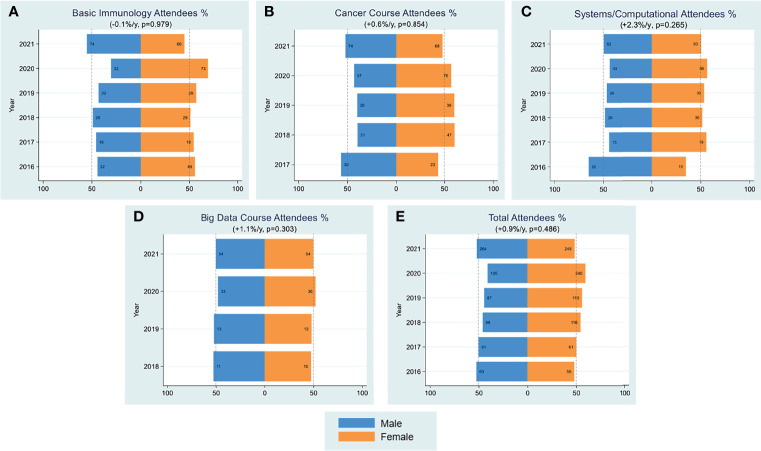
Attendees Workshop. Yearly female percentages of attendees in workshop on subject of basic immunology **(A)**, cancer **(B)**, systems/computational **(C)**, big data **(D)** and all categories combined **(E)** are shown over the period 2016-2021. Percentages were based on actual numbers subjects stating their gender. Average changes per year and p-values (shown in parentheses) were estimated by the slope coefficient from a simple linear regression model. The cancer and big data courses were initiated in 2017 and 2018, respectively. The dotted line represents 50% as a point of reference for parity.

**Figure 4:**
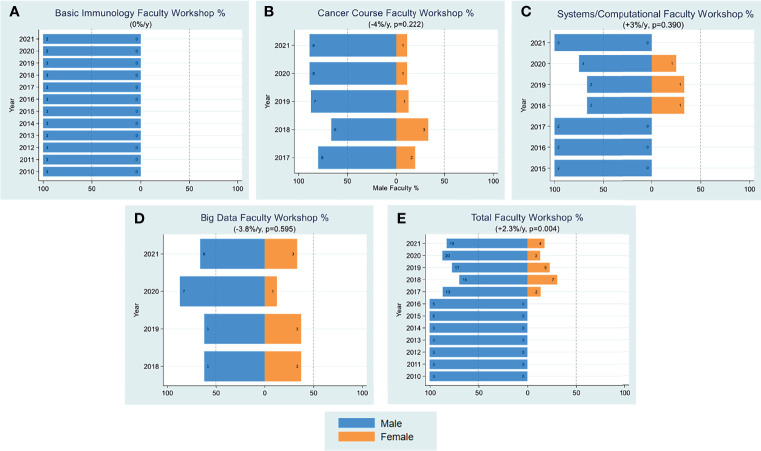
Faculty Workshop. Yearly female percentages of faculty in workshop on subject of basic immunology **(A)**, cancer **(B)**, systems/computational **(C)**, big data **(D)** and all categories combined **(E)** are shown over the period 2010-2021. Percentages were based on actual numbers subjects stating their gender. Average changes per year and p-values (shown in parentheses) were estimated by the slope coefficient from a simple linear regression model. The systems/computational, cancer, and big data courses were initiated in 2015, 2017 and 2018, respectively. The dotted line represents 50% as a point of reference for parity.

**Figure 5:**
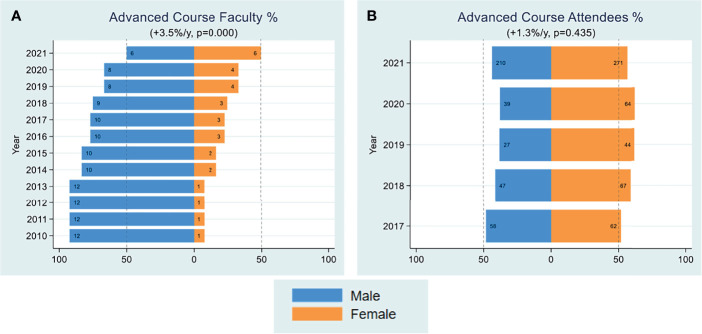
Advanced Course. Yearly female percentages of faculty in advanced course **(A)**, attendees in advanced course **(B)** are shown over the period 2010-2021. Percentages were based on actual numbers subjects stating their gender. Average changes per year and p-values (shown in parentheses) were estimated by the slope coefficient from a simple linear regression model. Gender data was not collected for advanced course attendees until 2017. The dotted line represents 50% as a point of reference for parity.

A major component of FOCIS’s mission is to organize educational workshops that provide educational opportunities for trainees and junior faculty, facilitate their interactions with experts in the field and build collaborations. FOCIS has sponsored courses and workshops in several areas, including basic immunology in medicine, cancer immunity and immunotherapy, systems immunology (formerly computational immunology) and big data in immunology. We assessed trends in attendance of men and women at FOCIS educational workshops (**Figure 3**) and faculty educators during 2016-2021 (**Figure 4**). Overall, women comprised about 55-60% of attendees across all of the courses (**Figure 3**). However, during the period of 2010-2017, only male faculty taught the FOCIS basic immunology in medicine course and educational workshops (**Figure 4**). To date, FOCIS has never had a female faculty member in the basic immunology in medicine course. From 2017-2021 there was an average of 2.3% per year increase in female faculty teaching at FOCIS educational workshops, but overall the number of male faculty has outweighed female educators (**Figure 4**). Female faculty teaching the advanced course in basic and clinical immunology were grossly underrepresented at <20% in 2010, <40% from 2015-2020, and finally achieved a 50% parity in 2021 (**Figure 5**). Underrepresentation of women in key educational roles and the lack of visible female role models can have profound negative implications for all trainees.

### Women Are Underrepresented in FOCIS Leadership Roles

**Figure 6:**
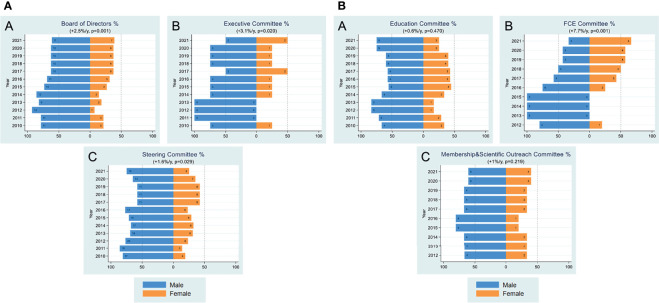
**(A)** Leadership. Yearly female percentages in board of directors (A), executive committee (B) and steering committee (C) are shown over the period 2010-2021. Percentages were based on actual numbers subjects stating their gender. Average changes per year and p-values (shown in parentheses) were estimated by the slope coefficient from a simple linear regression model. The dotted line represents 50% as a point of reference for parity. **(B)** Leadership. Yearly female percentages in education committee **(A)**, FCE committee **(B)**, membership & scientific outreach committee **(C)** are shown over the period 2010-2021. Percentages were based on actual numbers subjects stating their gender. Average changes per year and p-values (shown in parentheses) were estimated by the slope coefficient from a simple linear regression model. Gender data for FCE and membership & scientific outreach committees were not collected until 2012. The dotted line represents 50% as a point of reference for parity.

The gender composition of the society’s leadership is interesting to consider for several reasons. As shown in **Figure 6A**, there has been a general upward trend since 2010 in the percentage of women on the executive committee, board of directors and the steering committee. While this trend is encouraging, the numbers of individuals in these groups are small. The executive committee includes only the president, the past president, the president-elect and the secretary/treasurer, and the 50% female representation in 2021 reflects the gender of the past president, Maria-Grazia Roncarolo, and of the president-elect, Megan Sykes. Dr. Roncarolo was the first female president of FOCIS and during her presidency established the goal of having alternating male and female presidents, thereby ensuring a range of 25-75% female representation at all times. Maintaining this goal moving forward is particularly important, as the society’s leaders have a strong voice in determining committee membership and meeting programs and also provide role models for potential future leaders.

The board of directors has had a solid female presence since 2015, where women have comprised about 30-40% of the membership (**Figure 6A**). It is expected that this trend will continue in the next few years to reach an average of 50% female representation. The steering committee, whose members are appointed by individual member societies, has fluctuated between about 20 and 40% women since 2010. With growing awareness of the need for gender inclusiveness and exemplified by changes in FOCIS itself, it is anticipated that there will be continued increases in male-female equality that is reflective of the membership of each society.

The composition of the major committees is also worth examining, as these committees have considerable impact on the FOCIS membership at large (**Figure 6B**). The education committee has been about 20-40% women since 2010 and the membership committee has been in approximately the same range, although both are small committees. The annual meeting program committee has been 20-40% women between 2010-2020 before increasing to 58% in 2021 and including the appointment of a female chair. Of interest, the FCE committee, which is a bit larger, shows a sustained increase in female participation since 2015, with 50% female membership in 2021. Efforts to maintain this balance will be impactful, as the FCEs are focused on engaging the younger generation, whose future behavior as leaders will likely be modeled on current leadership. A leadership roster listing the total number of male and female board and committee positions over time is shown in **Table 1**.

**Table 1:** Leadership Rosters.

Year	2010	2011	2012	2013	2014	2015	2016	2017	2018	2019	2020	2021
**Board of Directors**												
**Total**	14	14	14	11	14	15	16	16	16	16	16	18
**Male**	79%	79%	93%	82%	86%	73%	69%	63%	63%	63%	63%	61%
**Female**	21%	21%	7%	18%	14%	27%	31%	37%	37%	37%	37%	39%
**Executive Committee**												
**Total**	4	4	4	4	4	4	4	4	4	4	4	4
**Male**	75%	100%	100%	100%	75%	75%	75%	50%	75%	75%	75%	50%
**Female**	25%	0%	0%	0%	25%	25%	25%	50%	25%	25%	25%	50%
**Steering Committee**												
**Total**	21	21	26	26	25	21	22	19	19	19	20	20
**Male**	81%	86%	77%	69%	68%	71%	77%	58%	58%	58%	65%	75%
**Female**	19%	14%	23%	31%	32%	29%	23%	42%	42%	42%	35%	25%
**Education Committee**												
**Total**	6	7	6	6	6	9	7	7	10	10	12	12
**Male**	67%	71%	83%	83%	67%	56%	57%	57%	60%	60%	75%	75%
**Female**	33%	29%	17%	17%	33%	44%	43%	43%	40%	40%	25%	25%
**FCE Committee**												
**Total**	0	0	5	5	5	5	8	7	8	7	7	6
**Male**	NA	NA	80%	100%	100%	100%	75%	57%	50%	43%	43%	33%
**Female**	NA	NA	20%	0%	0%	0%	25%	43%	50%	57%	57%	67%
**Membership & Scientific Outreach Committee**												
**Total**	0	0	6	6	6	5	5	6	6	6	10	10
**Male**	NA	NA	67%	67%	67%	80%	80%	67%	67%	67%	60%	60%
**Female**	NA	NA	33%	33%	33%	20%	20%	33%	33%	33%	40%	40%
**Annual Meeting Scientific Program Committee**												
**Total**	7	7	8	11	12	9	11	8	10	10	11	12
**Male**	57%	43%	50%	73%	83%	78%	82%	75%	60%	60%	64%	42%
**Female**	43%	57%	50%	27%	17%	22%	18%	25%	40%	40%	36%	58%
**All Committee Chairs**												
**Total**	7	7	7	7	7	7	7	7	7	7	7	7
**Male**	71%	71%	86%	86%	100%	86%	71%	71%	14%	29%	29%	71%
**Female**	29%	29%	14%	14%	0%	14%	29%	29%	86%	71%	71%	29%

### Female Underrepresentation in Member Society Symposia Faculty

**Figure 7:**
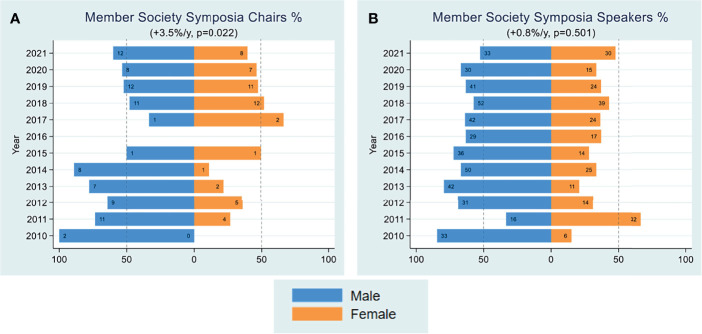
Member Society Symposia Data. Yearly female percentages of member society symposia chairs **(A)** and member society symposia speakers **(B)** are shown over the period 2010-2021. Percentages were based on actual numbers subjects stating their gender. Average changes per year and p-values (shown in parentheses) were estimated by the slope coefficient from a simple linear regression model. Gender data for member society symposia chairs was not collected for 2016. The dotted line represents 50% as a point of reference for parity.

FOCIS was established as a federation of disease- and organ-specific societies that have an interest in interdisciplinary and translational immunology as a component of their main area of practice/study. FOCIS has grown to 60 member societies that are invited to host symposia at the FOCIS annual meeting either independently or collaboratively with other members societies. We analyzed trends for women invited as member society symposia chairs and speakers. Women consistently represented 40% of the invited symposia speakers across the past 11 years (average 0.8% increase per year), and there was a notable increase in the percentage of female symposium chairs at an average rate of +3.5%/year during the same time frame (**Figure 7**).

### Survey Responses

A 12-question survey for feedback on FOCIS’s gender equity policies and practices was distributed to past and present leadership, steering committee members, senior and junior members, and management staff (**Supplement Table 2**). Among the 131 FOCIS participants invited to participate, 45 responded (34.3% response rate): 27 men (88 invited, 30.7% response rate) and 18 women (43 invited, 41.9% response rate). A total of 82.2% had been involved with FOCIS for >6 years, with the majority having served as FCE directors (57.8%), or board (44.4%) or steering committee (33.3%) members, and 55.6% were involved in academic research. Approximately 95% of respondents found gender equality extremely or very important to the success of FOCIS and immunology, and 75% indicated that FOCIS has been very or somewhat successful at increasing gender equality and female leadership in the organization and in immunology.

In response to the question of whether FOCIS provides equal opportunities across all genders to advance in the organization, 62.8% replied in the affirmative, but 9.3% responded No, with follow-up comments including “there remains a traditional bias towards men, specifically friends of men involved in FOCIS” and “FOCIS does not have a culturally and structurally supportive environment for inclusion of women in leadership and committee activities”. While some respondents were able to detail steps taken by FOCIS to address gender inequalities, it was concerning that 50% of respondents indicated that they were unaware of strategies being implemented to increase gender equality in leadership and participation. As such, 57.5% of respondents agreed that additional strategies remain necessary to increase gender equality. Suggestions included improving existing strategies since consideration of gender balance is not always reflected in outcomes, and that directed selection is necessary.

Among the 10 FOCIS current, future and past presidents who were interviewed by email, 8 responded (80% response rate): 6 men (8 invited, 75.0% response rate) and 2 women (2 invited, 100% response rate). All who responded indicated that they were acutely aware of the gender imbalance in various committees (**Table 2**). Indeed, one respondent noted that “equitable representation of genders in organizations and scientific societies doesn’t happen automatically, but is the result of a systematic effort and a concerted action by the board and the leadership. Although individual members and individual past presidents may have tried hard to establish more gender balance in the past, a systematic team effort at the leadership level has been lacking.”

**Table 2:** Quotes from FOCIS presidents.

“FOCIS was wrestling with getting to a sustainable identity/constituency and financial model…. Diversity strategy might well have accelerated success in these transitions (different perspectives and constituencies to deepen the problem solving), but attention to this strategy was considered a distraction.”
“We were incredibly aware of these issues from the time FOCIS was founded, and spent a significant amount of time discussing these issues.”
“I think we need to acknowledge that it is a systemic problem. …while current graduate school classes are gender-balanced, faculty ranks still lag behind a lot”.
“Although individual members and individual past presidents may have tried hard to establish more gender balance in the past, a systematic team effort at the leadership level has been lacking.”

It is useful to seek insights into why gender balance was so difficult to attain. Several responders noted that despite lengthy discussion, the gender inequity that is generally present amongst senior scientists and clinician scientists led to a “small pool to begin with”, i.e. FOCIS was at the mercy of university-based disparities. There was also the observation that the organization initially lacked clear policies that ensured transparency in how leadership appointments were made, leading to an “*ad hoc*” process without broad and representative discussion.

It was also noted that “FOCIS leadership nominations required a track record with involvement in Programming, Abstract, or FCE committees before people are asked to serve on the Board, or to join a Leadership role and therefore the pool of candidates is relatively small.” As discussed above, women were underrepresented on most FOCIS committees which disadvantaged them for leadership nominations. As a consequence, the nominating committee was skewed toward men, which may have resulted in more male than female nominations. This unconscious bias towards self-selection has long been recognized as a barrier to change towards a more equitable seat-at-the-table (1).

An interesting perspective from one past president was that gender inequality may have been harder to overcome because of the relatively new state of the organization and federation model. Efforts to solidify the FOCIS brand and develop a sustainable financial model were a major focus for several years, possibly leading to diminished time/resources to take action on gender inequality. Interestingly, it was noted that in retrospect, the creation of a diversity strategy might actually have accelerated the success of the organization, but at that time, diversity strategies were considered a distraction. Finally, there was a comment that the federation model meant that the gender composition of the steering committee was not within the direct control of FOCIS.

Taken together, all respondents were aware of gender inequality and yet it took 12 years (2003-2015) for the FOCIS board to nominate a female president, Dr. Maria Grazia Roncarolo, and to develop an intentional plan for systemic change.

## Discussion

Professional societies play an essential role in enabling the research community to network and communicate scientific discoveries with colleagues in academia, industry and funding agencies. Recent studies have shown that unequal gender representation in societies can privilege certain society members and conference attendees over others (2). Disparities such as more male speakers at conferences, and more men in leadership roles within the society, can send a negative message to members that the environment is unwelcoming and raise concerns of bias (3–7). The impact of female underrepresentation in such roles has a lasting effect on the future by propagating the perceptions and unconscious biases that have contributed to the existing imbalance in gender representation in STEM disciplines and science awards all the way up to and including Nobel prizes (8–10). Therefore, it is essential for societies to provide a framework of policies for expected conduct to ensure equal benefit across all gender identities (5–7, 11–15).

The opportunity to take international leadership roles and present research to broad audiences is critical for career progression (16). Underrepresentation of female speakers and pervasiveness of so-called “manels” at conferences is no longer acceptable, as it perpetuates inequality that results in fewer opportunities for recognition and networking, and makes it more difficult to progress through the traditional academic promotion and tenure process. This in turn decreases the pool of senior female candidates for leadership and speaker opportunities, and the number of role models for junior researchers of both genders. Thus, although achieving gender equality in the academic clinical immunology community requires change at many levels, as an academic society, FOCIS has a key role in instigating change.

In order to quantify the degree of gender inequity in FOCIS, we undertook a comprehensive study to review 10 years of available gender data. This review of FOCIS participation and leadership has been illuminating. We found that whereas women consistently represent half the membership of FOCIS, they were underrepresented in almost all FOCIS-related committees, including those involved in planning the annual meeting, the board of directors and the steering committee. Women were also found to be a minority in all areas of FOCIS education. Leadership should represent the community it serves (17, 18). Our survey of past, present and future leadership and of the membership revealed broad awareness of the disparity in female inclusion in FOCIS activities and leadership for many years, leading to urgent calls-to-action for parity. An inflection towards parity at all levels of the FOCIS organization began to appear around 2016.

Women were severely underrepresented in planning the FOCIS annual meeting and, perhaps consequently, as session chairs and invited speakers (<30%) prior to 2015. The participation of women as speakers and chairs was approximately 40% in 2017, 2018 and 2021, an encouraging improvement but still falling short relative to the ~50% female meeting registrants. Women have been underrepresented to the point of exclusion from some FOCIS educational faculties, and they have been underrepresented in the board of directors, steering committee and, until recently, the executive committee. This lack of professional visibility can have profound consequences (19), including the perpetuation of the ‘Matilda effect’ that systematically under-recognizes the accomplishments and contributions of women, and the ‘leaky pipeline’ that results in a dramatic reduction in the proportion of women compared to men surviving each step up the research ladder. The keen awareness of the pervasive gender disparity appears to have prompted a change within FOCIS, as female participation has shown some encouraging trends in this period, including increases in program committee, speaking and chairing roles at the annual meeting and in leadership.

The awareness of gender inequality at FOCIS has resulted in several further positive steps to address the problem. The evaluation of gender balance metrics as has been done within this article is an important early step. The establishment of a women’s committee at FOCIS, WICI, will assure concrete efforts to advance the careers of women in our field be sustained. The selection of two female presidents in the past 5 years is another major step forward that provides momentum for further attention to this issue.

### Recommendations to Improve Gender Equality

We suggest a number of steps that can and should be taken by FOCIS to improve gender equality:

1. Educate existing and incoming leadership on the importance of considering and actively promoting emerging women in the field and organization, and in providing the same opportunities for advancement, representation and visibility in the organization that are afforded to men. This education can be provided through interactions at executive committee, nominating committee, program committee, board and steering committee meetings, in which gender equality should be a regular consideration in all nominations. A database of female FOCIS members indicating area of expertise, academic rank, major accomplishments and prior contributions to FOCIS and other immunology societies would go far in bringing names of potential female speakers, chairs and leaders to the attention of the relevant committees during their deliberations. Construction of such a database should be a high priority for FOCIS.
2. Formally establish metrics that highlight potential disparities and frequently report on this data to organizational decision-makers so that these can be addressed on an ongoing basis. These metrics should be provided at all FOCIS meetings and when nominating decisions are made about leaders, speakers and chairs. Defining and collecting key gender equality performance measures for FOCIS will be necessary for identifying and monitoring the targets for positive change (20).
3. Embrace programmatic solutions (networking, education, mentorship, coaching and professional development opportunities) that prepare women for leadership and other roles in the organization. The establishment of WICI provides an opportunity for the development of such solutions and such activities are in their mandate. FOCIS should commit to these activities by assuring a regular seat on the board of directors for the WICI Chair and providing needed support, including fundraising, for these programmatic solutions.
4. Continue to welcome, cultivate and encourage open and honest discussion of gender bias and actively explore the means to mitigate this. Several recent studies show that education and diversity training can improve implicit associations about women in science. For example, evidence emerged indicating the efficacy of the “Scientific Diversity” workshop, such that participants were more aware of gender bias, expressed less gender bias, and were more willing to engage in actions to reduce gender bias two weeks after participating in the intervention compared with two weeks before the intervention (21, 22). Similarly, a recent study evaluated the impact of diversity training on university faculty by assessing changes in implicit associations and explicit attitudes toward women in science. Study findings suggest that participation in a brief diversity training can improve implicit associations about women in science (22).
5. Permeate the culture with even more visible and meaningful evidence of the organization’s commitment to diversity, equity and inclusion. Specify in the organizational values, goals and governing documents, create events that are welcoming and clearly open to all in the organization, and make structurally apparent; to this end a working group (WICI) focused on gender issues has been developed. Creating a vision of the change process and developing strategies to bring the vision to fruition is critically important, as this will help establish FOCIS’s ability to retain and attract new members (23, 24). For example, FOCIS can set policies on gender equality including goals for gender ratios for organizational positions, and develop strategies to achieve these goals (25, 26).
6. Establish more actionable policies and procedures that promote gender parity throughout the organization. Studies have shown that conferences lacking codes of conduct risk creating and perpetuating negative environments that make underrepresented groups feel unwelcome (14). FOCIS conferences can include codes of conduct that address identity-based discrimination and sexual misconduct, provide mechanisms for anonymous impartial reporting, and contain clear consequences. These efforts will improve inclusivity and reduce the loss of scientists who have been historically marginalized. FOCIS can also institute policies to enhance speaker gender balance, to provide support for speakers with family responsibilities, and to actively monitor gender-related trends to achieve the equitable representation of female invited speakers (7). Maintaining gender balance among speakers has also been shown to increase productivity by catalyzing an exchange of ideas among a broader and more diverse pool of scientists (27).
7. Reflect on gender equality matters in strategic planning initiatives. Make it part of the long-term planning conversation. FOCIS should continuously measure the outcomes of its gender equality initiatives by monitoring member demographics and member satisfaction. FOCIS should implement mechanisms to collect key demographics (i.e. gender, age, ethnicity, professional discipline) and report on its diversity variables to leadership and members annually. FOCIS has the opportunity to make a global impact on gender equality through its member societies. Thus, FOCIS should work collaboratively with member societies to develop and implement organizational diversity and equality policies and assess and report outcomes annually.

As the leading organization for translational immunology, it is imperative for FOCIS to continue to grow and support a culture of inclusiveness across the organization. Opening a dialogue and avenues to pursue this goal are essential for the success of the society and we are now entering a new era working toward this goal. Although, we face many challenges, the changes that are occurring, including notable improvements in leadership composition and a dedicated committee to women in immunology, are just the beginning. Looking forward, we envision an exciting new future for the organization that embraces both a diverse and inclusive leadership that is reflective of its membership.

## Data Availability Statement

The datasets presented in this article are not readily available because authorization from FOCIS to release survey data is required. Requests to access the datasets should be directed to CM (email address: cmutrie@focisnet.org).

## Ethics Statement

Ethical review and approval was not required for the study on human participants in accordance with the local legislation and institutional requirements. The patients/participants provided their written informed consent to participate in this study.

## Author Contributions

ER, AC, ML, CM, TL, MR, and MS designed the research study, collected and analyzed the data and wrote the manuscript. All authors contributed to the article and approved the submitted version.

## Conflict of Interest

The authors declare that the research was conducted in the absence of any commercial or financial relationships that could be construed as a potential conflict of interest.

## Publisher’s Note

All claims expressed in this article are solely those of the authors and do not necessarily represent those of their affiliated organizations, or those of the publisher, the editors and the reviewers. Any product that may be evaluated in this article, or claim that may be made by its manufacturer, is not guaranteed or endorsed by the publisher.
