# Supplementary material for: The Women of FOCIS: Promoting Equality and Inclusiveness in a Professional Federation of Clinical Immunology Societies
Source: Front Immunol. 2022 Apr 4;13:816535. doi: 10.3389/fimmu.2022.816535 (PMC9015160; doi:10.3389/fimmu.2022.816535)
Supplement: Supplementary file 1 [file Table_1.docx]

**Supplementary Data Tables**

Supplement Table 1. Unreported/Missing Data

| **Supplement Table 1. Unreported/Missing Gender Data**  **Percentages = (# of missing/total)** | | | | | | | |
| --- | --- | --- | --- | --- | --- | --- | --- |
| **Year** | **2015** | **2016** | **2017** | **2018** | **2019** | **2020** | **2021** |
| **Annual Meeting Registrants** | 34.08%  (274/804) | 14.29%  (121/847) | 23.04%  (153/664) | 18.96%  (201/1060) | 44.57%  (431/967) | 4.13%  (51/1236) | 5.07%  (49/966) |
| **Attendees – Basic Immunology Course** | N/A | 13.25% (11/83) | 25.53% (12/47) | 16.18% (11/68) | 52.34% (56/107) | 3.67% (4/109) | 2.19% (3/137) |
| **Attendees – Cancer Course** | N/A | N/A | 20.9% (14/67) | 15.22% (14/92) | 47.15% (58/123) | 6.38% (9/141) | 1.39% (2/144) |
| **Attendees – Systems or Computational** | N/A | 15.69% (8/51) | 17.07% (7/41) | 13.43% (9/67) | 29.11% (23/79) | 9.17% (10/109) | 3.1% (4/129) |
| **Attendees – Big Data** | N/A | N/A | N/A | 16.00% (4/25) | 39.00% (16/41) | 1.43% (1/70) | 5.26% (6/114) |
| **Attendees - Total** | N/A | 14.18% (19/134) | 21.29% (33/155) | 15.08% (38/252) | 43.71% (153/350) | 5.59% (24/429) | 2.86% (15/524) |
| **Advanced Course in Basic & Clinical Immunology**  **ATTENDEES** | N/A | N/A | 0.83% (1/121) | 18.57% (26/140) | 37.17% (42/113) | 25.9% (36/139) | 31.97% (226/707) |
| **Student/Trainee Membership** | 23.55% (61/259) | 19.57% (109/557) | 20.32% (90/443) | 27.05% (132/488) | 22.57% (107/474) | 29.43% (171/581) | 29.56% (175/592) |
| **Regular Membership** | 23.48% (54/230) | 22% (55/250) | 21.9% (60/274) | 23.08% (72/312) | 23.48% (81/345) | 24.3% (96/395) | 25.1% (126/502) |
| **Industry Membership** | 39.13% (18/46) | 31.08% (23/74) | 37.04% (30/81) | 51.96% (53/102) | 45.16% (42/93) | 33.33% (26/78) | 29.47% (28/95) |
| **Total Membership – All Categories Combined** | 24.86% (133/535) | 21.23% (187/881) | 22.56% (180/798) | 28.49% (257/902) | 25.22% (230/912) | 27.8% (293/1054) | 27.67% (329/1189) |

Supplement Table 2. Gender in FOCIS and Immunology Survey

| **Supplement Table 2. Gender in FOCIS and Immunology Survey** | | |
| --- | --- | --- |
| **Q1. How do you identify your gender?** | | |
| **Answer Choices** | **Responses** | |
| Woman | 40% | 18 |
| Man | 60% | 27 |
| Other | 0% | 0 |
|  | **Answered** | **45** |
|  | **Skipped** | **0** |
| **Q2. What is your age?** | | |
| **Answer Choices** | **Responses** | |
| Under 25 | 0% | 0 |
| 25-29 | 0% | 0 |
| 30-39 | 4.44% | 2 |
| 40-49 | 15.56% | 7 |
| 50 years and above | 80% | 36 |
|  | **Answered** | **45** |
|  | **Skipped** | **0** |
| **Q3. How long have you been involved in FOCIS?** | | |
| **Answer Choices** | **Responses** | |
| Less than 1 year | 4.44% | 2 |
| 1-2 years | 6.67% | 3 |
| 3-5 years | 6.67% | 3 |
| 6-10 years | 20% | 9 |
| Over 10 years | 62.22% | 28 |
|  | **Answered** | **45** |
|  | **Skipped** | **0** |
| **Q4. I most closely identify my professional role as (select all that apply):** | | |
| **Answer Choices** | **Responses** | |
| Academician | 55.56% | 25 |
| Clinician | 17.78% | 8 |
| Researcher | 60% | 27 |
| Graduate Student | 0% | 0 |
| Fellow | 2.22% | 1 |
| Medical Student | 0% | 0 |
| Industry Representative | 6.67% | 3 |
| Government Employee | 4.44% | 2 |
| Other (please specify): | 11.11% | 5 |
|  | **Answered** | **45** |
|  | **Skipped** | **0** |
| **Q5. I currently hold or have previously held the following roles in FOCIS  (please select all that apply):** | | |
| **Answer Choices** | **Responses** | |
| Board Member | 44.44% | 20 |
| Steering Committee Member | 33.33% | 15 |
| Committee Chair | 24.44% | 11 |
| Committee Member | 37.78% | 17 |
| Member Society Representative | 22.22% | 10 |
| FCE Director | 55.56% | 25 |
| Member | 57.78% | 26 |
| Not Applicable | 2.22% | 1 |
| Other (please specify): | 15.56% | 7 |
|  | **Answered** | **45** |
|  | **Skipped** | **0** |
| **Q6. How important is the issue of gender equality to the success of FOCIS and in immunology?** | | |
| **Answer Choices** | **Responses** | |
| Extremely important | 64.44% | 29 |
| Very important | 31.11% | 14 |
| Somewhat important | 4.44% | 2 |
| Slightly important | 0% | 0 |
| Not at all important | 0% | 0 |
| Don't know | 0% | 0 |
|  | **Answered** | **45** |
|  | **Skipped** | **0** |
| **Q7. How would you rate the success of FOCIS's efforts to increase gender equality and increase female leadership and participation in the organization and in immunology?** | | |
| **Answer Choices** | **Responses** | |
| Extremely successful | 6.67% | 3 |
| Very successful | 40% | 18 |
| Somewhat successful | 35.56% | 16 |
| Slightly successful | 6.67% | 3 |
| Not at all successful | 0% | 0 |
| Don't know | 11.11% | 5 |
|  | **Answered** | **45** |
|  | **Skipped** | **0** |
| **Q8. In FOCIS, are opportunities to advance in the organization (leadership positions, speaking opportunities, etc.) equal across all genders?** | | |
| **Answer Choices** | **Responses** | |
| Yes | 62.79% | 27 |
| Sometimes | 27.91% | 12 |
| No | 9.30% | 4 |
| If you answered no, please specify what is lacking: | | 5 |
|  | **Answered** | **43** |
|  | **Skipped** | **2** |
| **Q9. Are you aware of strategies that are being implemented to increase gender equality in leadership and participation in FOCIS and in immunology?** | | |
| **Answer Choices** | **Responses** | |
| Yes | 50% | 22 |
| No | 50% | 22 |
| If you answered yes, please specify what those strategies are: | |  |
|  | **Answered** | **44** |
|  | **Skipped** | **1** |
| **Q10. Is the implementation of additional strategies necessary to increase gender equality in leadership and participation in FOCIS and in immunology?** | | |
| **Answer Choices** | **Responses** | |
| Yes | 57.50% | 23 |
| No | 42.50% | 17 |
| If you answered yes, please specify what those strategies are: | |  |
|  | **Answered** | **40** |
|  | **Skipped** | **5** |
| **Q11. Do barriers exist to gender equality in leadership and participation in FOCIS and in immunology?** | | |
| **Answer Choices** | **Responses** | |
| Yes | 41.86% | 18 |
| No | 58.14% | 25 |
| If you answered yes, please specify what those barriers are, and what can be done to eliminate these barriers? | | 17 |
|  | **Answered** | **43** |
|  | **Skipped** | **2** |
| **Q12. Do you have any additional comments on gender equality in FOCIS and in immunology?** | | |
|  | **Answered** | **17** |
|  | **Skipped** | **28** |
